# Oxidative stress in Wernicke’s encephalopathy

**DOI:** 10.3389/fnagi.2023.1150878

**Published:** 2023-05-16

**Authors:** Jun-Dong Wei, Xueming Xu

**Affiliations:** ^1^Department of Basic Medical Science, Medical College, Taizhou University, Taizhou, China; ^2^Department of Psychiatry, Taizhou Second People’s Hospital, Taizhou, China

**Keywords:** thiamine deficiency, oxidative stress, Wernicke’s encephalopathy, liver injury, neurodegenerative diseases

## Abstract

Wernicke’s encephalopathy (WE) is a severe life-threatening disease that occurs due to vitamin B1 (thiamine) deficiency (TD). It is characterized by acute mental disorder, ataxia, and ophthalmoplegia. TD occurs because of the following reasons: insufficient intake, increased demand, and long-term drinking due to corresponding organ damage or failure. Recent studies showed that oxidative stress (OS) can damage organs and cause TD in the brain, which further leads to neurodegenerative diseases, such as WE. In this review, we discuss the effects of TD caused by OS on multiple organ systems, including the liver, intestines, and brain in WE. We believe that strengthening the human antioxidant system and reducing TD can effectively treat WE.

## 1. Introduction

Wernicke’s encephalopathy (WE) is a severe neurological disorder characterized by ophthalmoplegia, acute mental confusion, and ataxia (Ota et al., 2020). It is caused by vitamin B1/thiamine deficiency (TD) that occurs due to the following reasons: insufficient nutrient intake, increased requirement, inadequate nutrient absorption from intestines, excessive deficiency, long-term alcohol consumption, or malignant tumors. Thiamine metabolites can be used as biomarkers for neurodegenerative diseases and thiamine supplements have a therapeutic effect on patients with neurodegenerative diseases, such as Alzheimer’s disease (AD), Parkinson’s disease (PD), and WE (Gibson et al., 2016). TD is mainly accompanied by organ failure and damage. The most common clinical symptom of WE is observed in malnourished alcoholics. TD is common in alcoholics primarily due to reduced food intake, storage, intestinal transport, and thiamine utilization caused by drinking (Subramanya et al., 2010). In TD, several functional changes occur in neurotransmission, of which the most significant is the toxic nervous excitatory state caused by glutamatergic and GABAergic systems (de Freitas-Silva et al., 2010). In addition, TD alters the pentose phosphate pathway, which impairs neural signaling due to reduced myelination of neurons. Moreover, it causes neuronal death in the diencephalon and cerebellum through severe damage to mitochondrial functions (Pannunzio et al., 2000).

Oxidative stress (OS) occurs due to an imbalance between reactive oxygen species (ROS) production and elimination (Hussain et al., 2016). Both free radical ROS and non-radical ROS play a critical role in regulating various physiological functions, such as host defense, cellular signaling, regulation of gene expression of the human metabolic processes and immune system (Chen et al., 2012; Lushchak, 2014). Under normal conditions, organisms can eliminate ROS. However, external factors, such as diet, radiation, pollutants, and lifestyle, can increase ROS (Tripathi et al., 2022), which can alter various cellular components, such as oxidation of proteins, DNA, and fats, and lead to related signaling pathways (Finkel and Holbrook, 2000; Bhattacharyya et al., 2014). OS caused by excessive ROS production can damage the liver and intestines, cause neurodegenerative diseases and cancer, and even affect the human life span (Finkel and Holbrook, 2000; Bhattacharyya et al., 2014; Patel, 2016; Kim and Sieburth, 2018) (Table 1).

In this review, we discuss the pathophysiological mechanisms of TD caused by OS in various organs, including the liver, intestines, and brain.

### 1.1. OS in liver injury

The liver is the most significant organ of the abdominal cavity. It plays a vital role in regulating numerous physiological functions, such as digestion, excretion, nutrient storage, metabolic homeostasis, synthesis of new substances, and breakdown of xenobiotic compounds (Trefts et al., 2017). Therefore, liver injuries and diseases, such as fatty liver, hepatitis, fibrosis, cirrhosis, and liver cancer, are severe health problems that threaten human life (Gravitz, 2014; Haga et al., 2015; Sia et al., 2017; Parola and Pinzani, 2019; Shabangu et al., 2020). In addition, exposure and ingestion of certain chemicals and industrial toxins, such as mycotoxins, aflatoxin, and hepatotoxins, damage the liver and pose a severe threat to human health (Grunhage et al., 2003). Immune liver injury, caused by an immune response, causes inflammatory cell infiltration and granuloma formation, as well as destructs the liver cell cord structure (Koyama and Brenner, 2017). Furthermore, long-term or heavy drinking can cause alcoholic liver injury and impair liver functions (Modi et al., 2017). Recent studies showed that OS plays a critical role in the molecular mechanisms of various liver injuries, such as liver fibrosis, non-alcoholic fatty liver disease (NAFLD), and hepatocellular carcinoma (HCC; Mortezaee and Khanlarkhani, 2018).

Thiamine is an essential vitamin and cofactor. It plays a vital role in carbohydrate metabolism as well as in numerous cellular metabolic processes inside the cytosol, mitochondria, and peroxidase, thereby leading to various clinical consequences (Marcé-Grau et al., 2019). Recently, studies have identified a relationship between chronic liver diseases and reduced serum thiamine concentration, and 80% of the patients with chronic alcoholism show TD (Thomson, 2000). Alcohol use or insufficient nutrition reduces thiamine storage, thereby causing TD. However, in patients with NAFLD, reduced intestinal vitamin absorption due to impaired metabolism, poor thiamine storage, decreased liver cell count, or impaired visceral blood flow is the primary cause of TD (Gu et al., 2022). In addition, chronic liver diseases gradually destroy the liver parenchyma, which results in metabolic defects of many nutrients and severe vitamin deficiency, for instance, TD. Additionally, recent studies showed that in patients with end-stage chronic liver failure, TD mainly occurs due to the depletion of thiamine reserves in the liver (Butterworth, 2009). In addition, chronic liver failure increases brain ammonia concentration (Hadjihambi et al., 2022). Moreover, TD decreases α-ketoglutarate dehydrogenase (*α*-KGDH) activity and induces oxidative damage in brain mitochondria, thereby increasing brain lactic acid release, OS, cell energy damage, and pro-inflammatory cytokines, which is consistent with the phenomenon in the brain of chronic end-stage liver failure (Pan et al., 2018). Further, in patients with end-stage chronic liver failure, the neuropsychiatric symptoms of patients with brain injury due to TD were not completely relieved after using ammonia reductant or liver transplantation (Butterworth, 2009). These findings indicate that timely and effective thiamine supplementation benefits patients with chronic liver failure.

ROS are highly active molecules that oxidize various biological materials, such as lipids, proteins, and nucleic acids. ROS-induced lipid peroxidation plays an essential role in apoptosis and autophagy (Su et al., 2019). The ROS-modified proteins have altered structures and functions that induce numerous diseases, including cancer, liver diseases, neurological disorders, and chronic inflammation (Jomova and Valko, 2011). NADPH oxidase (NOX) uses NAD(P)H to generate superoxide from oxygen, hence it is an essential source of ROS (Bedard and Krause, 2007). Previous studies showed that NOX1 and NOX4 mainly exist in the hepatocytes (Lan et al., 2015). Chronic alcohol consumption increases NOX4 expression in mitochondria and induces liver injury. Interestingly, pretreatment with GKT 137831, a NOX4 inhibitor, can reverse liver fibrosis and hepatocyte apoptosis by attenuating ROS production (Jiang et al., 2012). Moreover, in an alcohol-induced liver injury mouse model, NOX4 knockout using siRNA reduced superoxide in mitochondria and increased the survival rate of cells (Sun et al., 2017). ROS production and OS induced by ethanol or acetaldehyde in mitochondrial dysfunction alter the permeability and transition potential of the mitochondrial membrane, and thereby promote the release of cytochrome c and other death-promoting factors to stimulate liver cell death (Song et al., 2022). While p38 mitogen-activated protein kinase (MAPK) activates the caspase-3 pathway in hepatocytes and thus improves cell death (Shang et al., 2018). Moreover, inhibiting the OS-related signaling pathways can effectively inhibit hepatocyte damage (Zhao et al., 2022). Furthermore, the Nrf2/ARE pathway may reduce ethanol-induced OS and liver injury (Ghanim and Qinna, 2022).

Long-term excessive alcohol intake is the main cause of alcoholic liver injury (ALI). In humans, alcohol dehydrogenase 2 (ADH2) and aldehyde dehydrogenase 2 (ALDH2) are critical enzymes that eliminate alcohol from the liver. ADH2 catalyzes the conversion of ethanol to acetaldehyde, which induces OS and mitochondrial dysfunction. ALDH2 further catalyzes the oxidation of acetaldehyde to acetic acid. In addition, ethanol can produce ROS through the cytochrome P-450 2E1 (CYP2E1) metabolic pathway, which causes mitochondrial dysfunction, induces the transition pore of mitochondrial permeability, and releases mitochondrial apoptosis factors, such as cytochrome c (Kaphalia and Calhoun, 2013). Cytochrome c further activates caspase-9 and caspase-3 apoptotic proteins and hence causes apoptosis of hepatocytes (Li et al., 2002). Furthermore, long-term drinking increases OS production through microsomal ethanol oxidation system (MEOS), which alters mitochondrial structure and function and causes cell death, thus leading to liver injuries (Acharya et al., 2021). Additionally, long-term excessive drinking produces high amounts of NF-κB in Kupffer cells, which promotes the production of inflammatory cytokines, such as tumor necrosis factor-α (TNF-*α*) and Fas ligand (FasL; Gao and Bataller, 2011). These cytokines bind to and stimulate their associated death receptors, such as tumor necrosis factor receptor-1 (TNFR1) and Fas receptor, which triggers apoptosis via caspase-8 activation; thus, triggering mitochondrial apoptosis and cell death (Ferreira et al., 2012). In all, alcohol overconsumption causes inflammation and liver cell death through the ROS signal pathway.

**Table 1:** The role of oxidative stress on multiple organ systems in thiamine deficiency.

Organ	OS inducer	Function
Liver	NOX1, NOX4	Promotes hepatocyte apoptosis (Jiang et al., 2012; Lan et al., 2015; Sun et al., 2017)
	ADH2	Induces alcoholic liver injure (Modi et al., 2017)
	CYP2E1	Causes mitochondrial dysfunction (Kaphalia and Calhoun, 2013)
	MEOS	Leads liver injuries (Acharya et al., 2021)
	Cytokines	Causes liver cells apoptosis (Gao and Bataller, 2011; Ferreira et al., 2012; Ezhilarasan, 2021; Li et al., 2021)
	COX-2	Promotes liver fibrosis progression (Yang et al., 2020)
	CCl4	Induces hepatocyte apoptosis (Chen et al., 2017; Liu et al., 2018)
intestines	Microbial pathogens	Causes chronic intestinal diseases (Aw, 2005)
	MTX	Increases intestinal inflammation (Aldhahrani, 2021)
	DSS	Affects intestinal barrier in homeostasis (Mees et al., 2020)
	Aluminum	Induces apoptosis of crypt cells (Eltahawy et al., 2017)
	Harmful bacteria	Leads epithelial barrier destruction (Rychlik and Barrow, 2005; Butcher et al., 2017)
	NOX	Increases barrier permeability (Han et al., 2022)
brain	HO-1	Induces neuronal loss (Calingasan and Gibson, 2000)
	Organic acids	Reduces *α*-KGDH activity (Wajner et al., 2020)
	eNOS	Increases the accumulation of peroxynitrite (Kruse et al., 2004)
	Ammonia	Promotes neurodegeneration (Norenberg et al., 2005; Natesan et al., 2016)

ROS, reactive oxygen species; NOX, NADPH oxidase; ADH2, alcohol dehydrogenase 2; CYP2E1, cytochrome P-450 2E1; MEOS, microsomal ethanol oxidation system; NAFLD, non-alcoholic fatty liver disease; COX-2, cyclooxygenase-2; CCl4, hepatotoxin; MTX, Methotrexate; DSS, dextran sulfate sodium; TD, thiamine deficiency; eNOS, endothelial nitric oxide synthase isoform.

In non-alcoholic liver injury (NLI), liver damage due to free radicals is a crucial factor leading to the disease. Therefore, eliminating excessive free radicals has become an important treatment strategy. Furthermore, hepatocytes secrete inflammatory factors, such as TNF-*α*, that induce a cellular inflammatory response, activate cysteine aspirating protease, and cause cell apoptosis (Ezhilarasan, 2021). In addition, interleukins (ILs) are important inflammatory cytokines that increase after OS and NF-κB activation through the PI3K/AKT-nuclear factor erythroid2-related factor 2 (Nrf2) pathway during NAFLD progression (Li et al., 2021). Cyclooxygenase-2 (COX-2) catalyzes the synthesis of prostaglandins (PGs) and plays a vital role in liver fibrosis progression by promoting inflammatory reactions and increasing the activation and proliferation of liver cells (Yang et al., 2020). Recent reports suggested that CCl4 is a hepatotoxin that causes OS and increases the expression of inflammatory cytokines, such as TNF-*α*, IL-1*β*, and IL-6, as well as promotes hepatocyte apoptosis and liver injury (Chen et al., 2017). Moreover, CCl4 induces liver injury by regulating mitochondrial membrane permeability, activating caspase-8, increasing intracellular ROS production, and leading to apoptosis (Liu et al., 2018). In all, many cellular enzymes are released into the blood in the potential mechanism of liver injury (Balogun and Ashafa, 2016).

Cholestatic liver injury is a neutrophil-mediated inflammatory reaction and neutrophil–induced OS causes liver cell death (Zhang et al., 2019). OS involves the bile acid signaling pathway that chemotactically recruits neutrophils during cholestasis causing liver damage (He et al., 2021). The sphingosine 1-phosphate receptor (S1PR) inhibits cellular ROS production and reduces neutrophil aggregation without interfering with the systemic immune status (Zhang et al., 2019). Furthermore, early growth response factor-1 (EGR-1) regulates the potential therapeutic targets of ROS for inhibiting inflammation caused by cholestasis (Kim et al., 2006). Therefore, strengthening the hepatocyte antioxidant system can control OS in neutrophils and thus treat cholestatic liver injury.

### 1.2. OS in intestinal absorption

Thiamine is synthesized only by bacteria, fungi, and plants and plays an essential role in the normal growth and development of organisms. Hence, it is a vital vitamin for humans and must be obtained from food (Yang et al., 2019). Free-thiamine uptake is accomplished by thiamine transporter 1 (ThTR-1) and 2 (ThTR-2), encoded by SLC19A2 and SLC19A3, respectively, which are transmembrane proteins expressed in small and large intestines (Marcé-Grau et al., 2019). Previous studies showed that human ThTR-2 is supposed to have an important role in intestinal absorption (Said et al., 2004; Subramanian et al., 2006). Mutations in ThTR-2 have been reported to be associated with thiamine metabolic dysfunction syndrome 2 (THMD2), and the clinical and imaging features of ThTR-2 mutant patients were similar to those of WE, indicating that the syndrome was caused by a genetic disorder of thiamine metabolism (Kono et al., 2009). Recent studies reported that prevalence of TD is significant in patients undergoing bariatric surgery (Kerns et al., 2015; Albaugh et al., 2021). In addition, due to decreased thiamine absorption, bariatric surgery accounts for a large proportion of WE cases (Kohnke and Meek, 2021). Excessive alcohol consumption has been reported to be associated with an essential risk factor for WE after bariatric surgery (Singh and Kumar, 2007; Oudman et al., 2018). Intestines are the primary organs for food digestion, absorption, and metabolism, and participate in various physiological functions, such as nutrient absorption, pathogen detection, and intestinal homeostasis (Segrist and Cherry, 2020). However, due to unavoidable exposure to microbial pathogens and foreign substances, intestines are also an essential source of ROS (Ballard and Towarnicki, 2020). Intestinal OS activates intestinal barrier dysfunction and plays a vital role in the early stage of intestinal injuries, such as triggering the immune imbalance and inflammatory response (Han et al., 2016; Yang et al., 2019). In addition, OS causes and promotes several intestinal diseases, such as intestinal infections, inflammatory bowel disease (IBD), ischemic intestinal injury, and colorectal cancer (Bhattacharyya et al., 2014). Therefore, regulating the ROS balance is crucial for treating related intestinal disorders.

In the intestines, OS occurs when the antioxidant defense system cannot quickly eliminate excess ROS from the human body. ROS can oxidize proteins, lipids, and DNA, and thus damage cells (Jakubczyk et al., 2020). In addition, OS can oxidize and destroy proteins, lipids, and DNA at the molecular level, thereby leading to intestinal diseases (Cervantes-García et al., 2020). Under OS, the oxidized state of glutathione and glutathione disulfide causes oxidative damage and apoptosis of intestinal epithelial cells (Jin et al., 2021). Moreover, exposure to microbial pathogens and xenobiotics generate ROS in intestines that cause various chronic intestinal diseases, such as IBD, intestinal infections, and colorectal cancer (Aw, 2005). In addition, ROS regulate inflammatory responses and play a critical role in the intestinal barrier in homeostasis, infectious diseases, and intestinal inflammation (Coant et al., 2010). Due to a lack of integrity and tight connections between the intestinal epithelial cells caused by ROS in ulcerative colitis (UC), the T cell transfer to the epithelial barrier leads to the production of inflammatory mediators and deterioration of mucosal damage (Wang et al., 2016; Wan et al., 2022). Methotrexate (MTX) is one of the most influential and widely used drugs for treating autoimmune and skin diseases. Nonetheless, MTX treatment causes intestinal inflammation in mice and increases apoptosis by elevating Bax and caspase-9 expression levels, while inhibiting Bcl-2 expression levels through OS (Aldhahrani, 2021). Similarly, ROS can be produced due to goblet cell depletion in the colon tissues of the dextran sulfate sodium (DSS)-induced UC mice model (Mees et al., 2020). Recent studies showed that the OS damage to rat intestinal Paneth cells due to aluminum and ionizing radiation alters the abnormal cell morphology, such as the expansion of crypt cavity and the erosion of villus, induces apoptosis of crypt cells, and causes bacterial invasion in Paneth cells (Eltahawy et al., 2017). The intestinal microbiota is a vital part of the intestinal environment and plays an important role in regulating intestinal health. Thus, when the homeostasis of intestinal flora is destroyed, numerous harmful bacteria proliferate and produce ROS in host cells, leading to OS, inflammatory response, apoptosis of intestinal cells, and epithelial barrier destruction. Recent studies showed that pathogenic bacteria, such as *Helicobacter pylori* and *Salmonella typhimurium*, cause OS and homeostasis imbalance in the intestines (Rychlik and Barrow, 2005; Butcher et al., 2017). ROS produced by NOX and mitochondrial ROS increase barrier permeability and inflammation (Han et al., 2022). In addition, ROS produced by symbiotic bacteria act on epithelial cells to cause intestinal infections that inactivate ubiquitin binding enzyme 12 (Ubc 12) and thus affect NF-κB activity (Kumar et al., 2007). Additionally, ROS-induced DNA mutations contribute to the progression of colorectal cancer (Mandal, 2017). Moreover, ROS contribute to colorectal carcinoma progression by inducing CYP2E1 and generating carcinogenic etheno-DNA adducts (Linhart et al., 2014). In conclusion, ROS damages the intestines by regulating the intestinal microenvironment, thereby leading to TD, which contributes to WE.

### 1.3. OS in brain nerve cell injury

TD is associated with focal neuronal loss and increases OS in the brain (Hazell et al., 2013). The resulting oxidative state and lactic acid accumulation cause blood–brain barrier abnormality. In addition, in patients with TD, some functional changes occur in the neurotransmitters, especially in the glutamate and GABAergic systems, that lead to toxic nervous excitability (de Freitas-Silva et al., 2010). Furthermore, alterations in the pentose phosphate pathway decrease the myelination of neurons and thereby damage neuronal signaling (Moskowitz and Donnino, 2020). Additionally, TD severely damages mitochondrial functions, leading to selective neuronal death in the diencephalon and cerebellum (Depeint et al., 2006). Moreover, the thiamine-dependent processes are crucial to brain glucose metabolism, loss of thiamine can lead to abnormal OS, inflammation, and neurodegeneration of AD patient (Chen and Zhong, 2013). In fact, the conversion of free thiamine to bioactive form thiamine pyrophosphate (TPP) is essential for cellular energy metabolism. Magnesium, as a necessary cofactor for the conversion of thiamine to TPP, participates in the activity of thiamine (Ott and Werneke, 2020). Without magnesium, thiamine cannot function properly. Moreover, magnesium also acts together with thiamine as an important cofactor to control several key metabolic enzymes related to glucose, protein metabolism, fatty acid, and ATP production. Recent research has shown that patients with a history of excessive alcohol consumption and a risk of WE should always check their serum magnesium levels (Coughlan et al., 2016). Magnesium is also a cofactor to transketolase (TK) and its administration to chronic alcoholic patients being treated with thiamine demonstrated a positive effect on erythrocyte TK activity (Peake et al., 2013). Recent reports indicated that in animal experiments under TD conditions, TK activity and hippocampal neurogenesis were significantly reduced, and TK was involved in thiamine mediated hippocampal neurogenesis (Zhao et al., 2014). Inflammation mediated by NLR family pyrin domain containing 3 (NLRP3) inflammatory bodies is known to be closely related to nervous system diseases and can be activated by OS (Minutoli et al., 2016). Recent studies showed that NLRP3 inflammatory bodies are significantly activated in the microglia of TD mice (Xu et al., 2021). However, treatment with phenylthiamine reduced mitochondrial ROS levels, thus inhibiting lipopolysaccharide (LPS) and ATP-stimulated NLRP3 inflammation in BV2 cells. These results indicate that ROS play a critical role in neuronal cell death during TD.

OS, such as accumulation of heme oxygenase-1 (HO-1) and superoxide dismutase in microglia, nitrotyrosine and 4-hydroxynonal in neurons, and induction of endothelial nitric oxide synthase (NOS) in vulnerable areas, particularly the thalamus, are significant neurological characteristics of TD (Calingasan and Gibson, 2000). Furthermore, OS reduces α-KGDH activity, which further causes mitochondrial damage and lactic acid accumulation in the brain. Additionally, mitochondrial damage and OS in patients with organic acidosis lead to neurologic dysfunction (Wajner et al., 2020). Moreover, exposure of nerve cells to ammonia leads to OS, which plays an essential role in the swelling of astrocytes in brain edema (Norenberg et al., 2005). Recent studies reported that TD increases ROS levels, expression of several NOS subtypes, and accumulation of peroxynitrite in the brain (Gibson and Zhang, 2002; Kruse et al., 2004). Chronic liver failure occurs due to increased NOS-I expression in neurons of rat brains, leading to α-KGDH inhibition in the tricarboxylic acid (TCA) cycle (Rose and Felipo, 2005). In end-stage chronic liver failure, TD or ammonia exposure inhibits α-KGDH, thereby resulting in slowing TCA circulation, brain lactate accumulation, and loss of crucial brain protein activities due to oxidative damage of mitochondria (Schliess et al., 2002; Zhao et al., 2009; Pan et al., 2018). Furthermore, liver failure changes glutamate neurotransmitters, thereby exposing the brain to high concentrations of ammonia and altering glutamate transport by transporters. In addition, acute liver failure and hyperammonemia decrease brain glutamate transporter activity and increase extracellular brain glutamate concentration through the OS pathway, leading to brain edema and cell death (Natesan et al., 2016).

## 2. Conclusion

TD alters the normal physiological functions of multiple organ systems, including the liver, gastrointestinal, and central and peripheral nervous systems, which further aggravates TD. This causes oxidative metabolic damage, inflammation, and degeneration in the brain, eventually leading to severe neurological diseases, such as AD, PD, and WE. We believe that effective antioxidation measures along with thiamine supplementation are beneficial for preventing and treating WE and other neurodegenerative diseases associated with TD in humans.

## Author contributions

All authors listed have made a substantial, direct, and intellectual contribution to the work and approved it for publication.

## Conflict of interest

The authors declare that the research was conducted in the absence of any commercial or financial relationships that could be construed as a potential conflict of interest.

## Publisher’s note

All claims expressed in this article are solely those of the authors and do not necessarily represent those of their affiliated organizations, or those of the publisher, the editors and the reviewers. Any product that may be evaluated in this article, or claim that may be made by its manufacturer, is not guaranteed or endorsed by the publisher.
